# Tuned MWCNT/CuO/Fe_3_O_4_/Polyaniline nanocomposites with exceptional microwave attenuation and a broad frequency band

**DOI:** 10.1038/s41598-022-13210-4

**Published:** 2022-06-10

**Authors:** Saeedeh-Sadat Afzali, Seyedeh Hoda Hekmatara, Jamileh Seyed-Yazdi, Seyed Mohammad Bagher Malek Hosseini

**Affiliations:** Department of Physics, Faculty of Science, Vali-E-Asr University of Rafsanjan, P.O. Box 77139-36417, Rafsanjan, Iran

**Keywords:** Metals and alloys, Engineering, Chemical engineering

## Abstract

In this study, novel quaternary MWCNT/CuO/Fe_3_O_4_/PANI nanocomposites were synthesized with three different weight ratios of CuO/Fe_3_O_4_/PANI to MWCNT (1:3), (1:4), and (1:5), where all of its components were synthesized separately and then combined in specific weight ratios. CuO/Fe_3_O_4_/PANI transmission electron microscopy (TEM) images revealed that most nanoparticles were in a CuO/Fe_3_O_4_ hybrid form, with a narrow size distribution uniformly dispersed in a polymer background. The TEM and scanning electron microscopy (SEM) images of the MWCNT/CuO/Fe_3_O_4_/PANI nanocomposite revealed that the MWCNT was uniformly coated with CuO/Fe_3_O_4_/PANI. All three nanocomposites samples demonstrated superior microwave attenuation performance in terms of reflection loss and absorption bandwidth. The minimum reflection losses for MWCNT/CuO/Fe_3_O_4_/PANI nanocomposites (1:3), (1:4), and (1:5) were 45.7, 58.7, and 85.4, 87.4 dB, respectively. The absorption bandwidths (RL ≤ −10 dB) of MWCNT/CuO/Fe_3_O_4_/PANI nanocomposites (1:3), (1:4), and (1:5) were 6, 7.6, and 6 GHz, respectively.

## Introduction

Carbon is widely used in many applications due to its good electrical and thermal conductivity, low density, good corrosion resistance, low thermal expansion, and low elasticity. Carbon is used as a resistive element to convert input microwaves into heat. Due to the electric dipoles’ response within them to the alternating electric field of input waves, most carbon allotropes exhibit excellent microwave absorption capabilities^1,2^. Magnetic materials can also absorb the microwave’s magnetic field by an analogous process. Among the wide range of magnetic materials, magnetite (Fe_3_O_4_) is highly attractive for its usage as a magnetic dissipater and improver of the microwave attenuation property of carbon nanotubes or carbon-based materials because of its low toxicity, good compatibility, high unpaired spin density, and strong spin polarization at room temperature^3–5^. Carbon/ferrite-based composites exhibit a better balance of electrical conductivity and magnetic susceptibility than individual magnetic or carbonaceous absorbents, making them promising as electromagnetic wave absorbers^6^. Additionally, numerous reports confirm that combining other metal oxides with magnetite enhances the absorption properties of the resulting hybrid nanoparticles. The incorporation of metal oxide hybrids into magnetic materials results in the development of conduction loss mechanisms, residuals, interfacial polarization, electron spin resonance, and a resonant domain wall, all of which contribute directly to improving microwave absorption properties^7–9^. Many reports indicate that hybridized magnetite/metallic or magnetite/semiconductor nanoparticles embedded in a conductive polymer or loaded on carbon materials, especially carbon nanotubes, is an effective strategy in producing microwave absorber composites with sound absorption performance^10–13^. Copper oxide (CuO), a semiconductor with a narrow gap, was used to decorate carbon materials such as carbon fibers, carbon black, carbon nanotubes, and graphene to fabricate efficient microwave absorbing composites^10,11^. Although the microwave absorption properties of copper oxides (CuO and Cu_2_O) as a hybrid with magnetite have not been studied, we hypothesized that it could improve the final CuO/Fe_3_O_4_ hybrid’s dielectric and magnetic absorption. On the other hand, coating the magnetic surface with conductive polymers with high microwave absorption properties results in improved impedance matching and inhibits the aggregation, corrosion, and magnetic phase transformation of nanoparticles by producing heat during the microwave absorption process.

The most significant polymer in the category of conductive polymers is polyaniline. It has high electrical conductivity with high polarizability (due to the presence of strong chemical bonds or localized charges), polarization relaxation, and conductive loss and is used to synthesize highly effective microwave absorber composites. Polymers can also cause a better dispersion of NPs and enhance the interfacial area and multiple interfacial reflections between individual nanoparticles. Polyaniline can uniformly conjugate with CNT’s surface and other substrates by representing surface tension and reactive chemical sites to bind with functional groups of CNTs, which results in a further increment in the interfacial area^14,15^. Polyaniline can also significantly optimize the impedance matching of EW. Thus, polyaniline can enhance electromagnetic wave absorption via various mechanisms^16–22^.

The present study synthesized a novel quaternary MWCNT/CuO/Fe_3_O_4_/PANI nanocomposite using a step-by-step approach, ensuring that the CuO/Fe_3_O_4_ hybrid nanoparticles were prepared using the optimal protocol to achieve the desired magnetic properties and size distribution. Then, aniline was polymerized on the surface of CuO/Fe_3_O_4_ hybrid NPs via in situ polymerization, forming a CuO/Fe_3_O_4_/PANI nanocomposite. The CuO/Fe_3_O_4_/PANI nanocomposite was then loaded onto the MWCNT surface in weight ratios of three, four, and five times that of MWCNT, resulting in MWCNT/CuO/Fe_3_O_4_/PANI (1:3), (1:4), and (1:5) nanocomposites. The MWCNT/CuO/Fe_3_O_4_/PANI composites were characterized via the following methods: An X-ray diffraction pattern was used to identify the crystalline phases of CuO and Fe_3_O_4_. Fourier transform infrared (FTIR) confirmed the successful polymerization of aniline on the surface of nanoparticles. The morphology of CuO/Fe_3_O_4_/PANI and MWCNT/CuO/Fe_3_O_4_/PANI nanocomposites were investigated by SEM and TEM. To this end, SEM and TEM images indicated a uniform distribution of CuO/Fe_3_O_4_ nanoparticles embedded in the polymer and a uniform loading of MWCNT with CuO/Fe_3_O_4_/PANI nanocomposite. Vibrating sample magnetometer (VSM) analysis presented the superparamagnetic properties of MWCNT/CuO/Fe_3_O_4_/PANI nanocomposites with high saturation magnetizations (40–60 emu/g).

Electromagnetic parameters of nanocomposites were calculated in the range of 8–18 GHz through a vector network analyzer. The reflection losses versus frequency curves were plotted based on electromagnetic parameters for all samples with different thicknesses. The results showed that all samples had a high reflection loss ranging from -50 to -87 dB and a broad absorption band exceeding 6 GHz.

In addition to the usual mechanisms of microwave absorption in magnetic and dielectric materials, the unique design of the MWCNT/CuO/Fe_3_O_4_/PANI composite with multiple interfaces and weight ratio optimization of each composite component results in a significant improvement in the composite’s microwave absorption property. MWCNT/CuO/Fe_3_O_4_/PANI composites have different dielectric permittivity and magnetic permeability interfaces (conductor/semiconductor or conductor/insulator or semiconductor/insulator and non-magnetic/magnetic). The accumulation of localized surface charge at the boundary of two materials with different dielectric constants leads to attenuation of the electric component of the incident wave. Moreover, the skin depth decreases due to creating current density at a non-magnetic/magnetic interface, and the incident wave’s magnetic field deteriorates^23^.

## Experimental

This study prepared a novel MWCNT/CuO/Fe_3_O_4_/PANI quaternary composite using a controllable method involving a three-step process. The composite’s constituents were prepared under controlled conditions and then combined to form the final nanocomposite at an optimal weight ratio. First, a hybrid CuO/Fe_3_O_4_ nanoparticle was synthesized using the optimal protocol to achieve the best superparamagnetic property. Afterward, nanoparticles were used as an initiator to polymerize aniline and form a CuO/Fe_3_O_4_/PANI nanocomposite. Finally, the MWCNT was used as a substrate to carry and stabilize the CuO/Fe_3_O_4_/PANI nanocomposite.

### Materials

The MWCNTs were supplied with diameters and lengths ranging from 20 to 40 nm and 5–15 μm, respectively, and a minimum purity of 95%. Other raw materials, including FeCl_2_.4H_2_O, FeCl_3_.6H_2_O, CuNO_3_ (purity > 98%), HCl, and NaOH (≥ 97%), were acquired from Sigma Aldrich. Aniline and ammonium persulfate (APS) were obtained from Merck.

### Preparation of CuO/Fe_3_O_4_ hybrid nanoparticles

To obtain a homogeneous solution, 1.51 g copper nitrate and 2.31 g iron oxide were mixed with 40 mL of deionized water and stirred. Then, 0.1 mol NaOH was added dropwise until the pH of the solution reached 13. Finally, the product was collected with a magnet, washed, and dried at 40 °C.

### Synthesis of CuO/Fe_3_O_4_/PANI nanocomposites

The CuO/Fe_3_O_4_/PANI nanocomposite was prepared via in situ polymerization of aniline on the functional groups of CuO/Fe_3_O_4_ NPs at a temperature lower than 4 °C in a 20 mL aqueous HCl solution (an oxidizing agent).

First, 1.5 g of prepared CuO/Fe_3_O_4_ NPs and 0.75 g of aniline monomer (1:2 ratio of aniline to CuO/Fe_3_O_4_) were dissolved in 20 mL aqueous HCl solution. Then, the mixture was exposed to ultrasonic waves for 30 min to disperse the NPs (separate nanoparticles in the clusters). The polymerization reaction must be performed at a temperature less than 4 °C. Afterward, 0.8 g of ammonium persulfate (oxidizing agent) was dissolved in 30 mL of deionized water and added dropwise to the mixture. Adding the first oxidizing droplets leads to the appearance of a green–blue color, indicating the beginning of polymerization. When the polymerization was complete, the products were collected by a magnet, and the solution was washed to remove the residual aniline and reach neutral pH^16–18^.

### Preparation of MWCNT/CuO/Fe_3_O_4_/polyaniline nanocomposites

To prepare the final nanocomposite, 0.5 g of CuO/Fe_3_O_4_/PANI nanocomposite and 0.1 g of MWCNT were dispersed separately in 100 mL of deionized water (the weight ratio of MWCNT to CuO/Fe_3_O_4_/PANI was (1:5)). Subsequently, two suspensions were added together and sonicated for 30 min. The sediment was then separated from the liquid by centrifugation. Finally, the black precipitate was dried in an oven at 50° C. Similar nanocomposites were prepared using weight ratios of MWCNT to CuO/Fe_3_O_4_/PANI of 1:4 and 1:3^16^.

The successful synthesis of MWCNT/CuO/Fe_3_O_4_/PANI nanocomposite was verified by determining its chemical bonding and crystalline planes using FT-IR (Nicolet IS10) and X-ray diffraction (XRD) (PANalytical), respectively. The magnetic behavior of products was investigated at room temperature in the presence of a magnetic field using a vibrating sample magnetometer (VSM JDM-13). The TEM technique was used to study the morphology of CuO/Fe_3_O_4_/PANI and MWCNT/CuO/Fe_3_O_4_/PANI nanocomposites using MIRA3 TESCAN and Tecnai. The composite’s electromagnetic properties and microwave absorption capability were determined using the transmission/reflection waveguide method on an Agilent vector network analyzer (VNA) E8364B over 8.2–18 GHz.

## Results and discussion

### Material characterization

**Figure 1:**
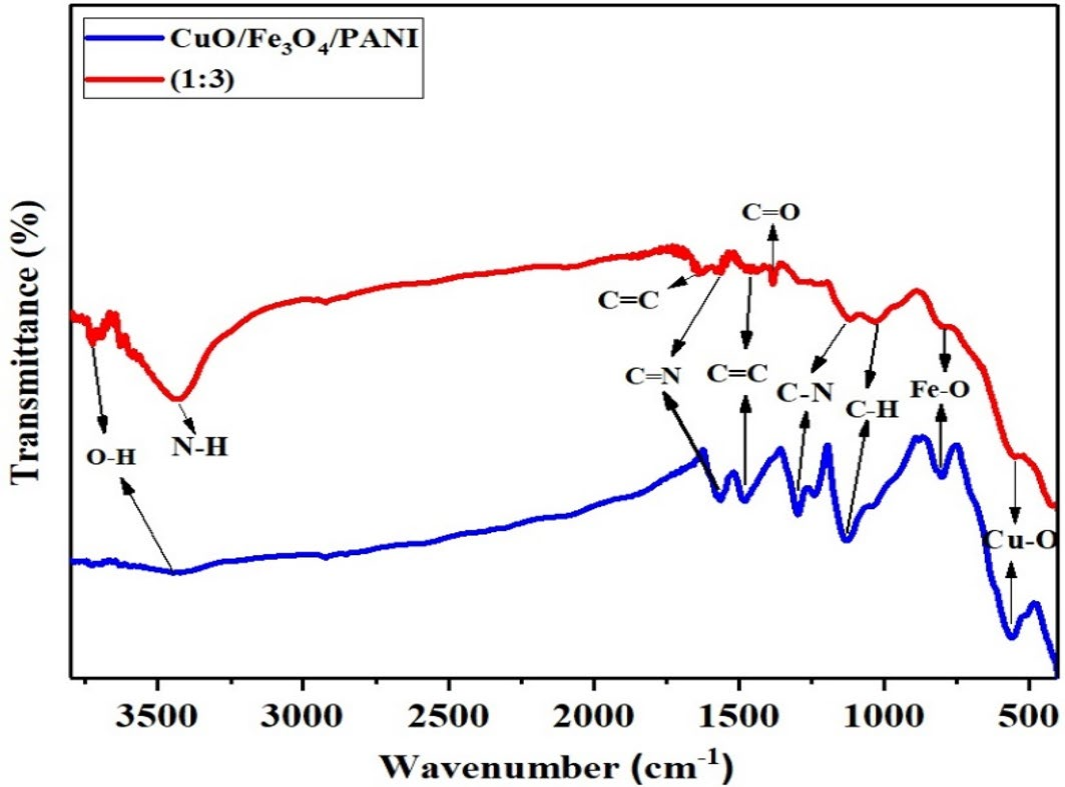
FT-IR spectra of CuO/Fe_3_O_4_/PANI and MWCNT/CuO/Fe_3_O_4_/PANI (1:3).

The FT-IR spectra of CuO/Fe_3_O_4_/PANI and MWCNT/CuO/Fe_3_O_4_/PANI nanocomposites were prepared by the KBr tablet method. According to Fig. 1, the broad signal at 3441 cm^−1^ in the CuO/Fe_3_O_4_/PANI spectrum is due to the presence of O–H groups and the carboxylic acid (C = O and C–O) functional groups of CNT at 1562 and 1150 cm^−1^. The absorption band at 1562 cm^−1^ also corresponds to the tensile vibration of C = N corresponding to the quinoid ring in the polyaniline structure. The characteristic peak of carbon nanotubes at 1638 cm^−1^ is related to the symmetrical expansion vibration of the C = C bonds. The absorption band at 1479 cm^−1^ is attributed to the C = C functional groups in the benzenoid rings in the polyaniline structure^24^. The absorption band at 1296 cm^−1^ demonstrates the tensile vibration of the C–H bonds, while the absorption band at 1131 cm^-1^ exhibits the bending vibration of the C-H bonds, which is a characteristic of polyaniline bonds^25^. The absorption band at 800 cm^−1^ is associated with the Fe–O functional groups, while the absorption band at 559 cm^−1^ is linked with the Cu–O functional groups. Peaks in the range of 800 cm^−1^ are characteristic of the substitution of aromatic rings, which indicates the formation of the polymer^26^. The absorption band at 3435 cm^-1^ is related to the tensile vibration of N–H bonds in polyaniline. The absorption band at 1570 cm^−1^ is due to the tensile vibration of the C = N bonds corresponding to the quinoid ring. The absorption bands at 1119 cm^−1^ and 1030 cm^−1^ represent the expansion state of C–N bonds and the bending vibration of C-H bonds, respectively.

**Figure 2:**
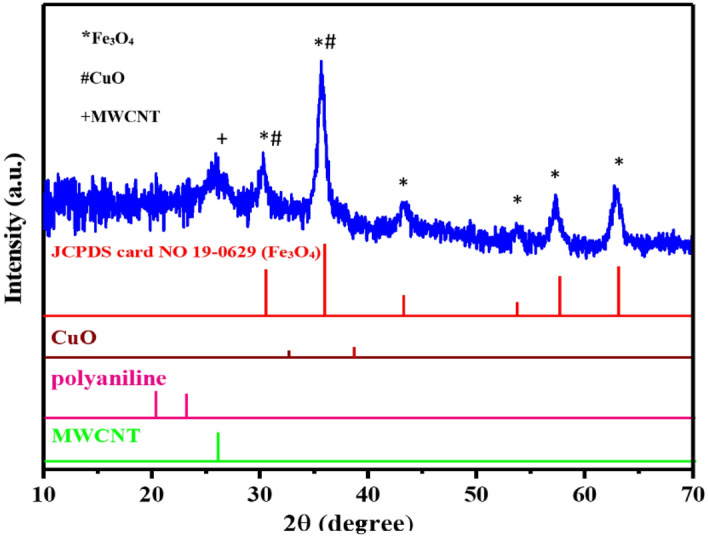
XRD pattern of MWCNT/CuO/Fe_3_O_4_/PANI (1:3).

The crystalline structure of MWCNT/CuO/Fe_3_O_4_/PANI nanocomposite was determined by the X-ray diffraction pattern. As illustrated in Fig. 2, the scattering peaks around 2θ = 30.3°, 35.6°, 43.4°, 53.5°, 57.3°, and 62.7° correspond to the Bragg plates (220), (311), (400), (422), (511), and (440), respectively, and are entirely consistent with the diffraction patterns of magnetite (Fe_3_O_4_). According to JCPDS Card 19–0629, this pattern corresponds to the face center cubic structure (FCC) of Fe_3_O_4_, and no additional peaks are observed to indicate the presence of impurities^27^. The peak of the carbon nanotube was observed at an angle of 25.9°, corresponding to the (002) of graphite’s hexagonal structure^26^. The peak at 39.4° is associated with the (200) and (111) planes, while the peak at 32.5° is attributed to the (110) plane, which is indexed to CuO’s monoclinic structure (JCPDS Card 48–1548)^27–29^.

**Figure 3:**
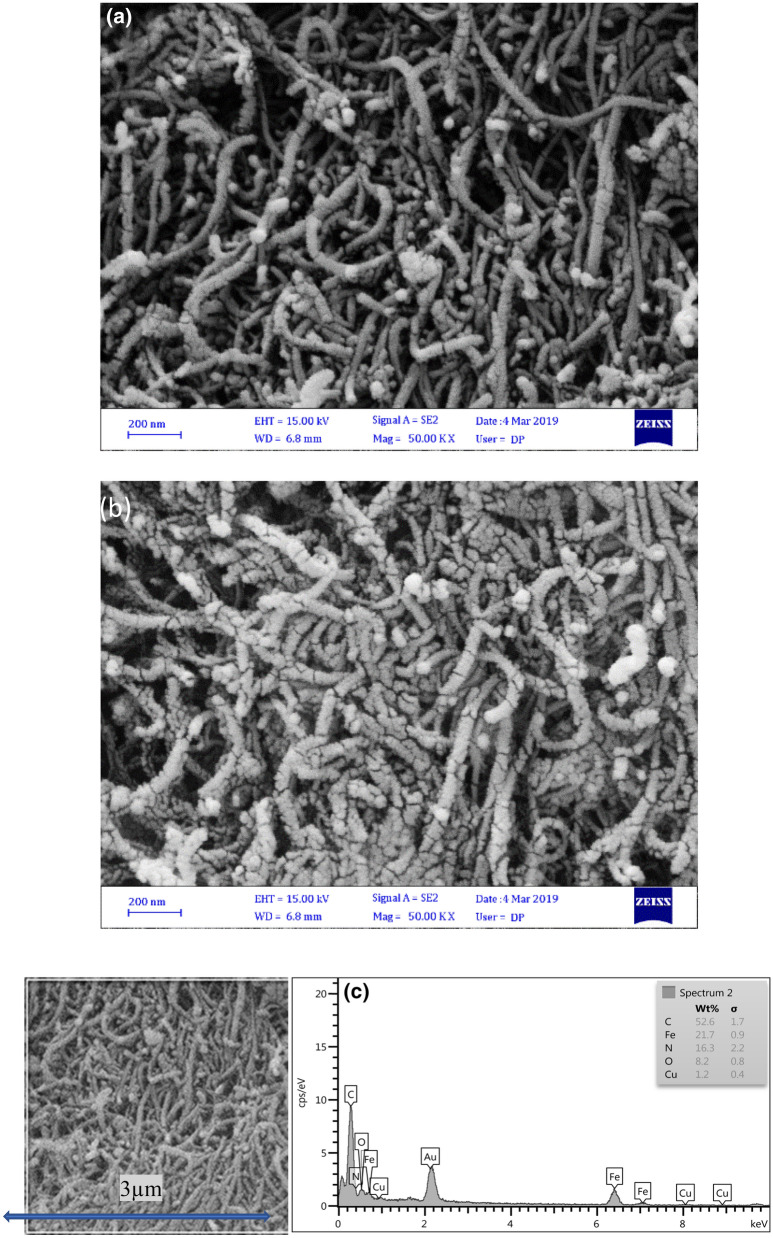
FESEM images of: (**a**) MWCNT/CuO/Fe_3_O_4_/PANI (1:3) and (**b**) (1:5). EDS pattern of: (**c**) MWCNT/CuO/Fe_3_O_4_/PANI (1: 3).

Field emission scanning electron microscopy (FESEM) was used to study the morphology of the synthesized powder of MWCNT/CuO/Fe_3_O_4_/PANI (1:3) and (1:5). FESEM images of MWCNT/CuO/Fe_3_O_4_/PANI (1:3) and (1:5) (Fig. 3, a & b) demonstrate that the OH groups of CuO/Fe_3_O_4_ NPs act as an initiator for aniline polymerization during the polymerization process. Thus, a polymer layer is formed on the surface of CuO/Fe_3_O_4_ NPs, resulting in nanoparticle separation within the polymer matrix. Because the activation energy required to form a polymer on the surface of CuO/Fe_3_O_4_ NPs is significantly lower than the activation energy required in the absence of such a surface, aniline tends to initiate polymerization spontaneously on such surfaces. The initial diameter of purchased nanotubes was 20 nm. After the polymerization process, the average diameter increased to 40 and 42 nm for MWCNT/CuO/Fe_3_O_4_/PANI (1:4) and (1:5) nanocomposite, respectively (Fig. 3a,b), indicating an almost uniform coating of CuO/Fe_3_O_4_/PANI nanocomposite on the MWCNT’s surface. The ends of the nanotubes contain more OH and COOH functional groups than the rest of their surfaces, which interact via hydrogen bonding with aniline’s amino groups. As a result, a few small clusters can be seen at the end of the CNTs in FESEM images. Electron diffraction spectroscopy (EDS) was performed to determine the type of elements present in a 9 µm^2^ surface area of MWCNT/CuO/Fe_3_O_4_/PANI (1:4) nanocomposites’ thin film (Fig. 3c). EDS diagram shows that the weight percentage of carbon, iron, nitrogen, oxygen, and copper are 52.6, 21.7, 16.3, 8.2, and 1.2, respectively.

**Figure 4:**
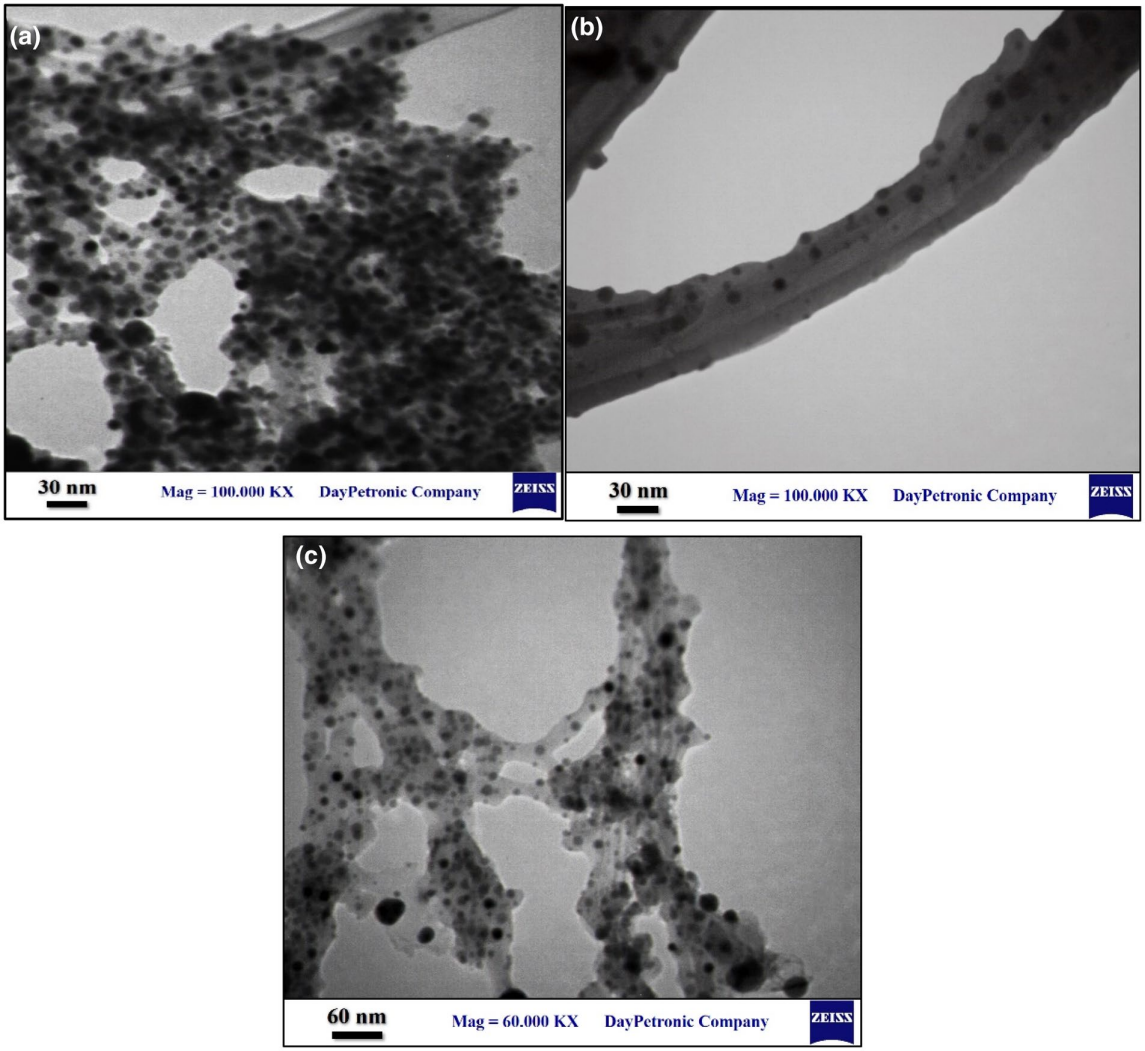
TEM images of: (**a**) CuO/Fe_3_O_4_/PANI, (b) MWCNT/CuO/Fe_3_O_4_/PANI (1:3) and (**c**) MWCNT/CuO/Fe_3_O_4_/PANI (1:5).

The TEM image of the CuO/Fe_3_O_4_/PANI nanocomposite (Fig. 4a) demonstrated that the CuO/Fe_3_O_4_ hybrid NPs exhibit a spherical shape, are predominantly core–shell with a black core, and are embedded in the polymer matrix with a narrow size distribution (4–6 nm). Additionally, a few isolated CuO NPs (gray) and Fe_3_O_4_ NPs (black) were observed. TEM images of MWCNT/CuO/Fe_3_O_4_/PANI nanocomposite (1:3) and (1:5) (Fig. 4b,c) demonstrate that the entire surface of the nanotubes was coated with a uniform thickness of about 40 nm of CuO/Fe_3_O_4_/PANI nanocomposite, indicating that the nanotubes were buried beneath the polymeric shell. Moreover, the polymer increases the specific surface area of the nanotubes through proper adhesion and reduces surface tension at the interface, in addition to chemical bonding to the functional groups of the CNT. These features contribute to the uniform adhesion of CuO/Fe_3_O_4_/PANI to MWCNT. No apparent aggregation of nanoparticles was observed in the TEM images. Interconnected polymer chains increase the solubility of nanotubes in the reaction medium and prevent them from merging and clustering without using the surfactant. As a result, coating polymer on the surface of CuO/Fe_3_O_4_ NPs causes uniform deposition of CuO/Fe_3_O_4_ NPs on the outer surface of CNTs, as shown in the TEM images (Fig. 4b,c).

## Magnetization and microwave absorption properties

**Figure 5:**
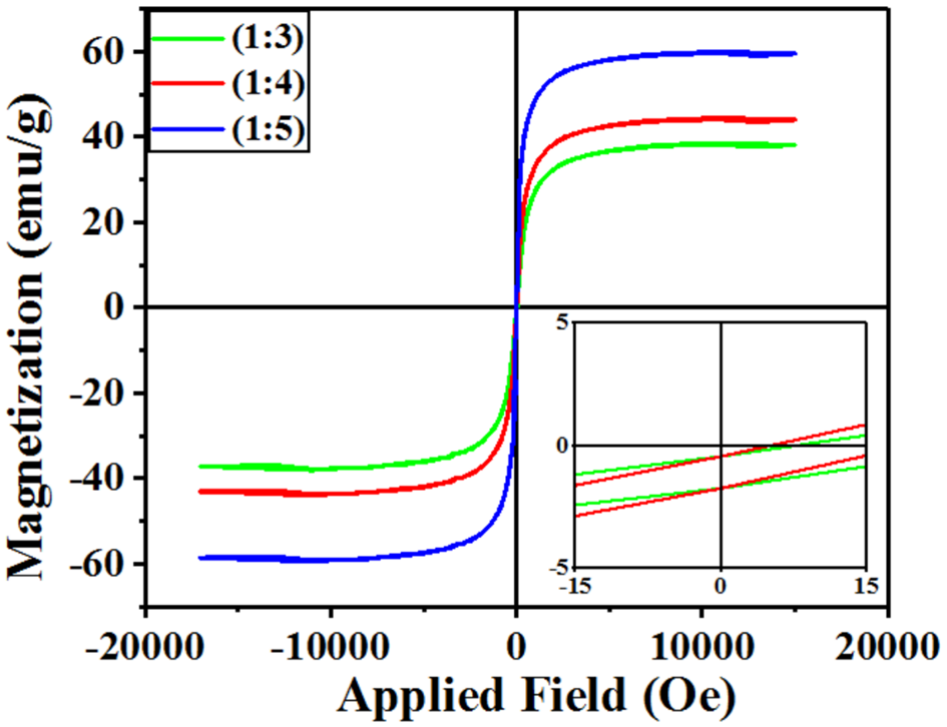
Magnetization versus applied magnetic field (Oe), at room temperature, for MWCNT/CuO/Fe_3_O_4_/PANI (1:3), (1:4) and (1:5).

As shown in Fig. 5, VSM analysis of different samples of MWCNT/CuO/Fe_3_O_4_/PANI was performed at room temperature (25 °C) in the range of -15,000 to 15,000 Oe and in the saturation magnetization (Ms) range of ± 60 emu/g. A closed-loop of hysteresis was observed for MWCNT/CuO/Fe_3_O_4_/PANI nanocomposites (remanences are lower than 5 emu/g and coercivities are below 15 Oe), indicating the presence of superparamagnetic CuO/Fe_3_O_4_ NPs. MWCNT/CuO/Fe_3_O_4_/PANI saturation magnetization (1:3), (1:4), and (1:5) are 38.61, 47.5, and 60 emu/g, respectively. Magnetic saturation was expected to increase as the weight ratio of magnetic CuO/Fe_3_O_4_ NPs increased^30,31^.

### Electromagnetic parameters

**Figure 6:**
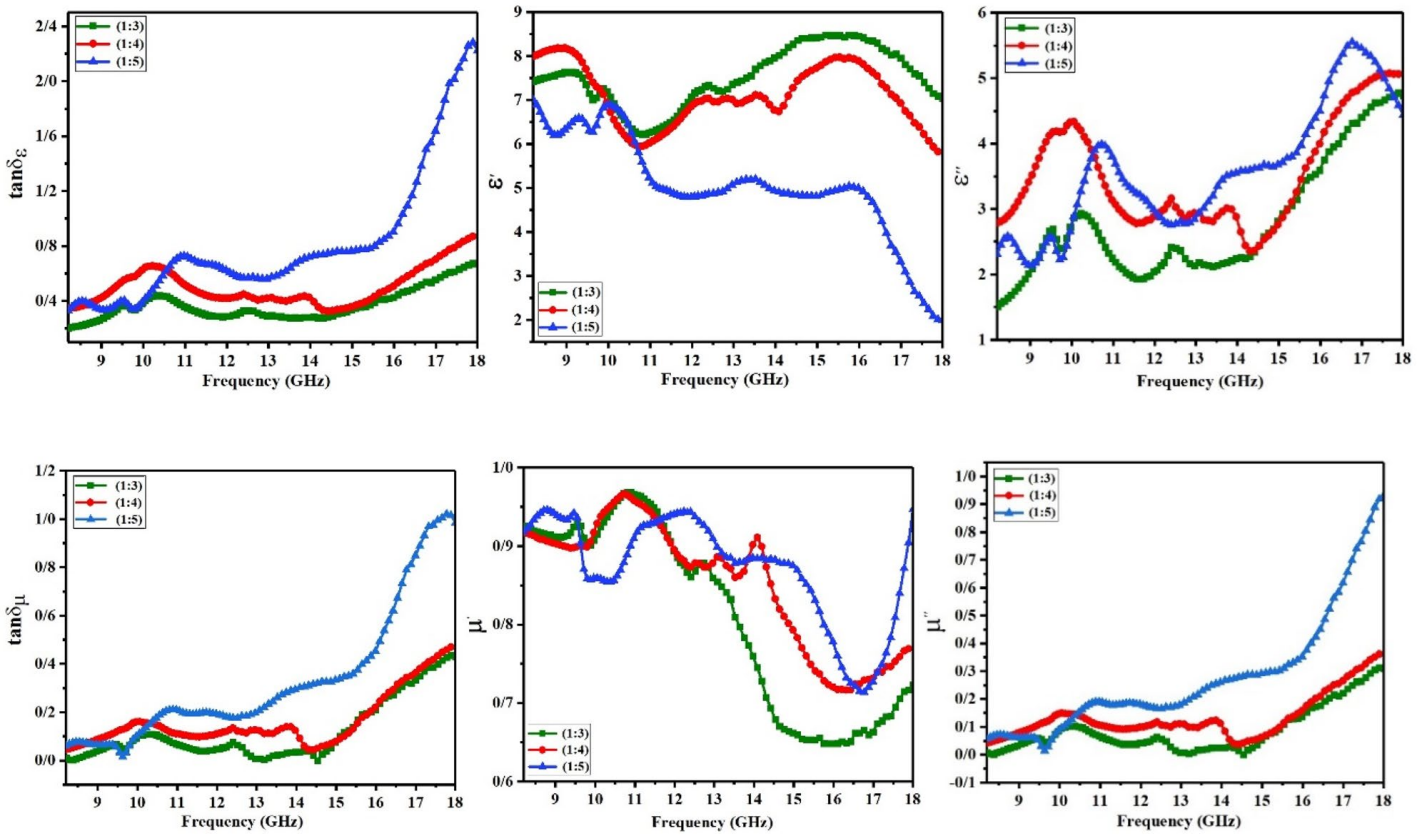
Up: Real and imaginary parts of permittivity and dielectric tangent losses of MWCNT/CuO/Fe_3_O_4_/PANI (1:3), (1:4), (1:5), Down: real and imaginary parts of permeability and magnetic tangent losses of them.

The electromagnetic (EM) parameters of MWCNT/CuO/Fe_3_O_4_/PANI nanocomposites were calculated to determine their absorption capacity. To this end, each nanocomposite was homogeneously dispersed in paraffin with a 25 wt% filler content. Complex permittivity (ε) and complex permeability (µ) are electromagnetic parameters that are intrinsic to each absorbent material and determine a matter's ability to absorb waves were achieved by a network vector analyzer (Anritsu.37269D) in the 8.2–18 GHz frequency range, presented in Fig. 6.

The real part of permittivity and permeability εʹ and µʹ, which show the degree of the polarizability of absorbent material, is a measure of the ability to store electrical and magnetic energy. εʺ and µ″, also known as dielectric and magnetic loss, refer to the attenuation of the incident wave’s electric and magnetic energy within the material, respectively^30,31^. εʺ and μ″, the imaginary parts, are related to polarization relaxation and spin rotation relaxation (aligning dielectric and magnetic dipole with alternative electric and magnetic fields of incident waves), respectively. εʺ originated from different polarization such as the electron, nuclei, dipoles, and interfacial polarization relaxation (space accumulated charge polarization). Here, space-charge polarization is the primary factor in the dielectric loss. This is due to additional polarizations at higher frequency ranges^32^. Interfacial polarization is caused by space-charge accumulation at the interface of materials with different dielectric constants. For conductive materials, εʺ typically originates from conduction loss as well as polarization relaxation^33^. From Fig. 6, it is clear that nanocomposite (1:3) exhibits the highest values of εʹ (6.2–8.5). This is characteristic of a high amount of CNT that demonstrates high values of εʹ and εʺ. Nanocomposite (1:4) has slightly lower values of εʹ and exhibits the same trend as nanocomposite (1:3). Nanocomposite (1:5) shows the lowest amount of εʹ because the weight percentage of CNT is the lowest one. The (ε) values of all samples of nanocomposites are almost the same. resulted from several factors: (i) charge accumulation at the interface of CuO and Fe_3_O_4_ in hybrid form, CuO or Fe_3_O_4_ in single form and polyaniline, CuO/Fe_3_O_4_ and polyaniline, polyaniline and CNT^34,35^. Dispersion of nanoparticles within the polymer results in a significant increase in the nanoparticles’ joint surface with the polymer. Additionally, the nanotube’s surface is coated with a polymer layer. The nanotubes’ high surface-to-volume ratio creates a large interface between the nanotubes and the polymer. It is well established that the larger the interface area, the more space charge, and polarization relaxation occurs^36^.

The presence of a conductive loss in polyaniline and CNT is another phenomenon that significantly contributes to the εʺ of nanocomposites. The polarization relaxation of the space charge at the NPs/PANI interface, which occurs at high frequencies (> 14 GHz), and the conductive loss of PANI appear to be the dominant mechanisms. In addition, multiple reflections occurred between separated CuO/Fe_3_O_4_ NPs, causing more incident wave dissipation^37,38^. As a result, the nanocomposite (1:5) with the highest amount of CuO/Fe_3_O_4_/PANI nanocomposite represents the highest εʺ and dielectric tangent loss (tanδ_ε_) at a higher frequency range.

**Figure 7:**
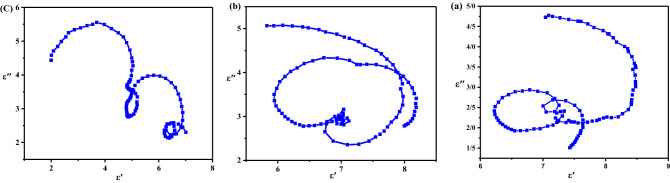
Typical Cole–Cole semicircles of :(**a**) MWCNT/CuO/Fe_3_O_4_/PANI (1:3), (**b**) (1:4) and (**c**) (1:5).

As illustrated in Fig. 6, the µʹ curves of all samples are similar. Except at the frequency extremes, the µʹ values of nanocomposites (1:4) and (1:5) are nearly identical, while the µʹ values of nanocomposites (1:3) are slightly lower. This observation is associated with the higher amount of Fe_3_O_4_ NPs in nanocomposites (1:4) and (1:5) than (1:3). µʹ demonstrates the possibility of magnetic dipoles in this regard. Since magnetic dipoles can only occur in magnetic materials with unpaired spin, by increasing the amount of magnetite, µʹ values increased. μ″ is related to spin rotation relaxation that initially corresponds to natural resonance in the ferromagnetic domain wall resonance, occurring at lower frequencies (< 100 MHz), exchange resonance, and eddy current^39^. our nanocomposites contain CuO/Fe_3_O_4_ with single domain superparamagnetic nanoparticles. Hence, exchange resonance and eddy current loss are involved in magnetic dissipation, occurring at high frequencies (> 13 GHz)^33^. Since ferrites have a relatively high electrical resistance^40^, they have a low dielectric loss and low eddy current loss. However, by incorporating CuO, a semiconductor with a narrow bandgap, to form a CuO/Fe_3_O_4_ hybrid, conductivity and eddy current loss may be increased. An increment in μ″ values and magnetic tangent loss (tanδ_μ_) of nanocomposite (1:5) over 13 GHz was expected. Generally, tanδ_ε_ is higher than tanδ_μ_ for all samples, indicating dielectric loss makes a larger contribution to microwave dissipation which has often been observed^41^. The relaxation (especially polarization relaxation) is an essential dielectric loss mechanism in the absorption of EM waves. The Cole–Cole model can be used to describe the relaxation process (Fig. 7). The Debye relaxation model is used to interpret ε scattering mechanisms. In this model, the Cole–Cole curve is presented in the shape of semicircles. Each semicircle represents a separate Debye relaxation. The radius of the semicircle exhibits relaxation time. The larger the radius, the longer the rest time. Long relaxation time corresponds to relaxation with higher periodicity and lower frequency^42^. Thus, resonance occurs at lower frequencies for these relaxations; vice versa, resonance occurs at higher frequencies for the smaller semicircle radius (short relaxation time).

In all three composites, one or two semicircles were observed, indicating multiple relaxation processes occur for the composites, proving the contribution of the Debye relaxation in increasing the dielectric dispersion of the composites^43,44^. The Cole–Cole curve of nanocomposite (1:3) is composed of large and small semicircles and indicates the presence of three relaxation resonances, two of which have long relaxation times and one of which has a short relaxation time. Nanocomposite (1:4) exhibits two large semicircles (with a higher radius than that of nanocomposite (1:3)) and a minute one which indicates two long relaxation times and a short one. Nanocomposite (1:5) shows two medium semicircles and two equal relaxation times. As per the statements above, polarization relaxation is a significant factor in the incident wave’s attenuation. The resonance peaks in the RL diagram depend on these relaxation frequencies. Thus, in nanocomposite (1:4) with the highest relaxation frequency, the minimum reflection loss is located at the lowest frequency, whereas in nanocomposite (1:3) to (1:5) with increasing relaxation frequency, the minimum reflection loss is located at higher frequencies.

### Reflection losses and absorption bandwidths

The reflection loss values for various sample thicknesses were calculated using the EM parameters:

1 $${Z}_{in}={Z}_{0}\sqrt{\frac{{\mu }_{r}}{{\varepsilon }_{r}}}\mathrm{ tanh}\left(j\frac{2\pi fd }{c}\sqrt{{\varepsilon }_{r}{\mu }_{r}}\right)$$

2 $$RL\left(dB\right)=20\, \mathrm{log}|\frac{{Z}_{in}-{Z}_{0}}{{Z}_{in}+{Z}_{0}}|$$

where *Z*_*in*_ is the input impedance of the microwave absorbing material, *Z*_*0*_ denotes the impedance of free space, *j* denotes the imaginary unit, *d* represents the thickness of the absorbent, *f* is the microwave frequency, *c* denotes the velocity of light, and RL_min_ is the reflection loss.

**Figure 8:**
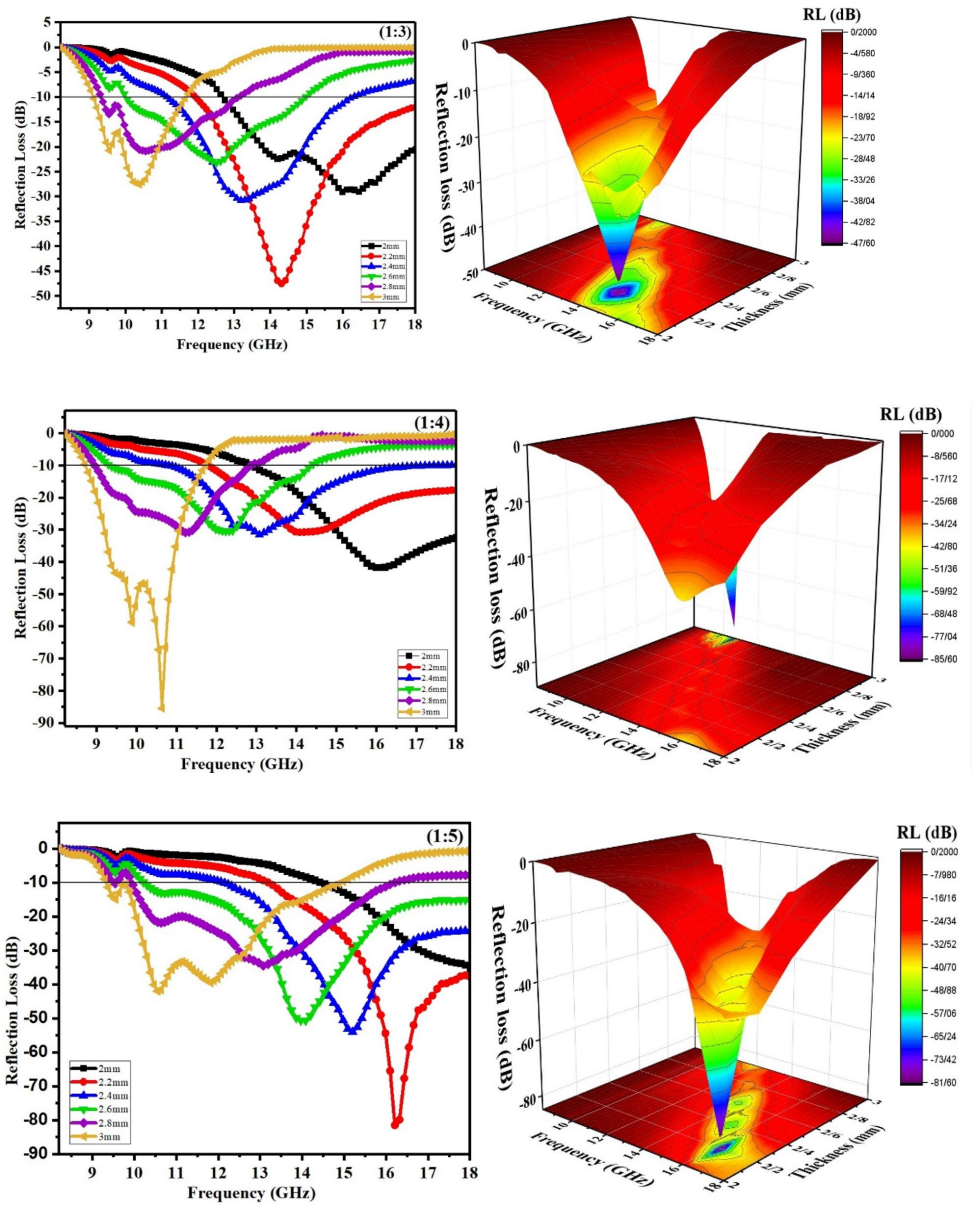
Reflection loss of MWCNT/CuO/Fe_3_O_4_/PANI (1:3), (1:4) and (1:5) versus frequency for different absorbent thickness (2D, 3D).

Figure 8 illustrates the reflection losses of MWCNT/CuO/Fe_3_O_4_/PANI (1:3), (1:4), and (1:5) versus frequency in the 8.2–18 GHz range. The results imply that all MWCNT/CuO/Fe_3_O_4_/PANI samples exhibit excellent MA behavior. The thickness of the absorber has a significant effect on the microwave absorption properties. The RL_min_ peak gradually moves to a lower frequency with increasing absorbent thickness, which follows the formula ${f}_{m}=c/2\pi \mu{''}d$, where ${f}_{m}$ is the frequency adapted to RL_min_, and *d* is the sample thickness. For all samples, two strong absorption peaks were observed at a thickness of 3 mm. Except for the thickness of 2.2 and 3 mm, both reflection loss and bandwidth decrease as the sample thickness increases in the nanocomposite (1:3) RL_min_ curve. A minimum reflection loss of −47.5 dB is achieved at 14.3 GHz with an absorbent thickness of 2.2 mm and an absorption bandwidth of approximately 6 GHz (12.2–18 GHz). For nanocomposite (1:4), the reflection loss decreases by increasing the sample thickness. For thicknesses of 3 mm, minimum reflection losses reach -85.4 and -58.7 dB at 10.6 and 9.8 GHz, respectively, with an effective bandwidth of 3 GHz (8.8–11.8 GHz). The broadest bandwidth is 7.6 GHz (10.4–18 GHz) for 2.2 mm absorbent. RL_min_ is greater than 87.4 dB at 16.2 GHz in nanocomposite (1:5), with the broadest absorption band of 6 GHz (12–18 GHz) for a 2.2 mm absorbent thickness. Two strong peaks with RL_min_ −45 and −43 dB appeared at 10.3 and 11.8 GHz, respectively, with the absorption band of 4.4 GHz (9.8–14.2) for a 3 mm absorbent thickness. The absorption bandwidths are greater than 3 GHz for all absorbent thicknesses.

**Figure 9:**
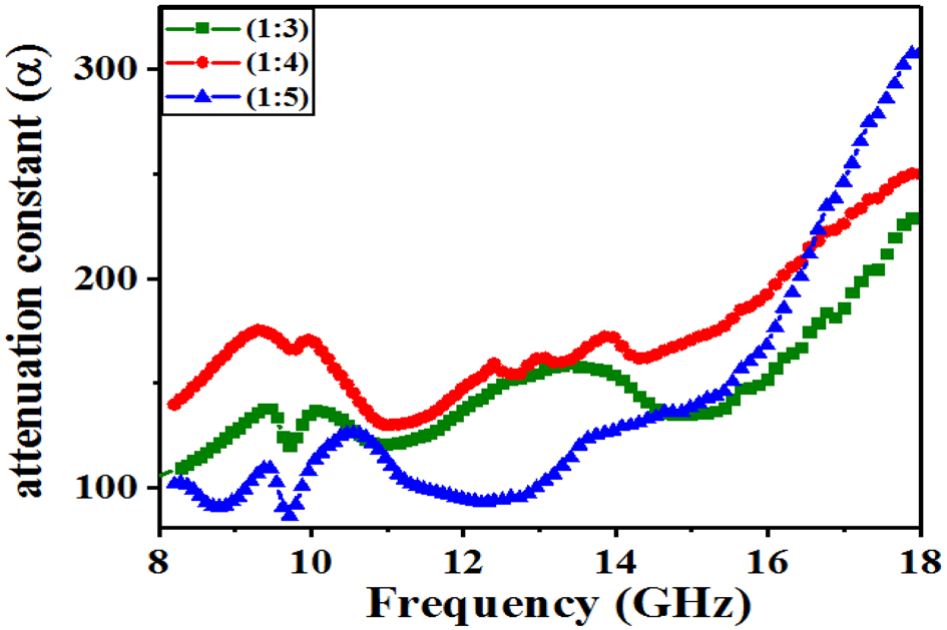
Attenuation constant (α) of MWCNT/CuO/Fe_3_O_4_/PANI (1:3), (1:4) and (1:5) versus frequency.

Figure 9 shows the loss constant (α) as the main effective factor in the range of microwave absorption. The loss constant (α) is used to evaluate the ability of integral absorption loss, calculated through the following formula:

$$\alpha =\frac{\sqrt{2} \pi f}{c}{[\left({\varepsilon }^{''}{\mu }^{''}-{\mu }^{^{\prime}}{\varepsilon }^{^{\prime}}\right)+\sqrt{[({{\varepsilon }^{''}{\mu }^{''}-{\varepsilon }^{^{\prime}}{\mu }^{^{\prime}})}^{2}+{\left({\varepsilon }^{''}{\mu }^{^{\prime}}+{\mu }^{''}{\varepsilon }^{^{\prime}}\right)}^{2}}]}^\frac{1}{2}]$$

The higher values (α) indicate the more powerful the magnetic and dielectric loss is if the microwave can reach the inner space of the absorber. The magnetic and dielectric loss tangent are two key factors influential in the absorption efficiency of an absorber. The higher values of loss tangent indicate the high ability to convert electromagnetic waves to other forms of energy. The attenuation constant was significantly increased in the 10–18 GHz frequency range. Meanwhile, the (1:5) exhibited a larger attenuation constant, which was also well consistent with the results of the RL curves.

Several absorption mechanisms discussed in Sect. 4.1 contributed to the remarkable absorption capacity of the MWCNT/CuO/Fe_3_O_4_/PANI nanocomposites, significantly greater than comparable composites reported in Table 1.

**Table 1:** Some reports from reflection losses and bandwidths of nanocomposites which similar to MWCNT/CuO/Fe_3_O_4_/PANI nanocomposites.

Filler	Matrix	Loading (wt%)	Thickness(mm)	Minimum RL (dB)	Broadest bandwidth (GHz) RL < -10 dB	Frequency range (GHz)	Ref
Fe_3_O_4_/PANI	Paraffin	50	2	−37.4	5.5	12.5–18	^45^
CNTs/PANI/Fe_3_O_4_	Paraffin	–	2	−22.4	6.9	6.4–13.3	^46^
CNTs/Fe_3_O_4_/PANI	Paraffin	20	2	−48	9.5	8.5–18	^47^
PANI/Fe_3_O_4_/MWCNTs	Resin	20	4	−16	7	8–15	^48^
Fe_2_O_3_/Fe_3_O_4_/MWCNTs	Paraffin	29	2.5	−44.1	3.3	8–12	^49^
Fe_2_O_3_/Fe_3_O_4_/PANI/CNT	Paraffin	25	3	−48.5	8.3	9.7–18	^16^
Fe_2_O_3_/Fe_3_O_4_/PANI/CNT	Paraffin	25	3.2	−80.8	5.3	9.2–14.5	^16^
MWCNT/CuO/Fe_3_O_4_/PANI (1:3)	Paraffin	25	2.2	−47.5	6	12.2–18	This work
MWCNT/CuO/Fe_3_O_4_/PANI (1:4)	Paraffin	25	2.2	−85.4	7.6	10.4–18	This work
MWCNT/CuO/Fe_3_O_4_/PANI (1:5)	Paraffin	25	3	−87.4	6	12–18	This work

## Conclusion

1. A multi-step method was prepared for a new quaternary MWCNT/CuO/Fe_3_O_4_/PANI nanocomposite.
2. CuO/Fe_3_O_4_ hybrid NPs with a narrow size distribution were prepared via a simple co-precipitation route.
3. The O–H functional groups on the surface of the CuO/Fe_3_O_4_ NPs served as initiators for the polymerization of aniline on the surface of the CuO/Fe_3_O_4_ NPs, resulting in the formation of a CuO/Fe_3_O_4_/PANI nanocomposite.
4. MWCNTs were loaded with different weight ratios (1:3), (1:4), and (1:5) of CuO/Fe_3_O_4_/PANI nanocomposite via simple physical agitation.
5. The FESEM and TEM images revealed that the CuO/Fe_3_O_4_ nanoparticles were dispersed in the polyaniline background with an almost uniform distribution. Moreover, the images demonstrated that MWCNTs were coated with a nearly uniform thickness of CuO/Fe_3_O_4_/PANI nanocomposite.
6. All MWCNT/CuO/Fe_3_O_4_/PANI samples had a superparamagnetic property with a high saturation magnetization ranging from 38 to 60 emu/g.

All MWCNT/CuO/Fe_3_O_4_/PANI samples exhibited superior absorption properties in terms of reflection loss and absorption bandwidth. Minimum reflection losses were 45, 58.7 and 85.4, 87.4 dB (2.2 mm absorbent) and 80 dB (3 mm absorbent) for MWCNT/CuO/Fe_3_O_4_/PANI (1:3), (1:4), and (1:5), respectively. The broadest absorption bandwidths were 6, 7.6 GHz (2.2 mm absorbent), and 6 GHz (2.6 mm absorbent) for MWCNT/CuO/Fe_3_O_4_/PANI (1:3), (1:4), and (1:5), respectively.
